# Y-stent-assisted coiling with pEGASUS stents for intracranial bifurcation aneurysms: A multi-center retrospective study

**DOI:** 10.1177/15910199251360143

**Published:** 2025-08-01

**Authors:** Abdallah Aburub, Ali Khanafer, Zakarya Ali, Mohammad Almohammad, Oussama Dob, Mete Dadak, Lars Timmermann, Ole Simon, Anja Gerstner, Mariana Gurschi, Yashar Aghazadeh, Christopher Nimsky, Benjamin Saß, Hans Henkes, André Kemmling, Stephan Felber

**Affiliations:** 1 Department of Neuroradiology, University Hospital of Giessen and Marburg, Philipps-University Marburg, Marburg, Germany; 2 Neuroradiology, 14881 Klinikum Stuttgart Katharinenhospital, Stuttgart, Germany; 3 Central Institute for Diagnostic and Interventional Radiology, Neuroradiology, and Nuclear Medicine, 9206 Sana Klinikum Offenbach, Offenbach, Germany; 4 Department of Radiology, St. Vincenz Hospital Paderborn, Paderborn, Germany; 5 Department of Neurology, University Hospital Marburg, Philipps-University Marburg, Marburg, Germany; 6 Department of Neurosurgery, University Hospital Marburg, Philipps-University Marburg, Marburg, Germany; 7 Department of Interventional Radiology and Neuroradiology, Rhön-Klinikum, Campus Bad Neustadt, Bad Neustadt an der Saale, Germany; 8 Institute for Diagnostic and Interventional Radiology and Neuroradiology, 39625 Stiftungsklinikum Mittelrhein, Koblenz, Germany

**Keywords:** Y-stent-assisted coiling, pEGASUS stent, intracranial aneurysms, endovascular treatment, bifurcation aneurysms, neurovascular intervention

## Abstract

**Objectives:**

Y-stent-assisted coiling (Y-SAC) is an established technique for managing wide-necked intracranial bifurcation aneurysms. However, data on the use of the pEGASUS stent, a self-expanding open-cell stent with an antithrombogenic hydrophilic polymer coating, remain limited. This study evaluated the effectiveness and safety of Y-SAC with pEGASUS stents in patients with intracranial bifurcation aneurysms.

**Methods:**

This retrospective observational study included patients treated with Y-SAC with pEGASUS stents at six neurovascular centers between July 2021 and June 2024. Data on aneurysm characteristics, procedural details, and clinical outcomes were collected. Aneurysm occlusion was assessed with the modified Raymond-Roy classification (MRRC) at 6 and 12 months. The primary endpoint was complete aneurysm occlusion (MRRC I), whereas secondary endpoints included perioperative complications, functional outcomes, and retreatment rates.

**Results:**

A total of 40 patients (mean age: 61.6 ± 9.4 years; 60% women) were included. Immediately post-procedure, 100% of aneurysms achieved complete occlusion (MRRC I). At 6–12 months follow-up, 92.5% maintained MRRC I occlusion, and 2.5% exhibited neck remnants (MRRC II). Functional outcomes were favorable in 95% of patients at discharge. The overall complication rate was 4.8%, and one patient (2.5%) required retreatment. No periprocedural thromboembolic events were observed.

**Conclusions:**

Our findings indicated that Y-SAC with pEGASUS stents achieves high rates of durable aneurysm occlusion with minimal complications, thus supporting its use as a safe and effective strategy for wide-necked bifurcation aneurysms. Future prospective studies are needed to validate long-term outcomes and optimize treatment strategies.

## Introduction

Intracranial bifurcation aneurysms, characterized by their occurrence at arterial branch points within the brain, pose substantial challenges in neurovascular intervention because of their complex anatomy and wide necks.^1,2^ Traditional endovascular treatments, such as simple coiling, often face limitations in effectively securing these aneurysms, thereby increasing risks of recurrence and procedural complications.^3^ To address these challenges, SAC techniques have been developed to provide enhanced support and facilitate more durable aneurysm occlusion.^4^

The Y-stent-assisted coiling (Y-SAC) approach has notably emerged as a valuable technique for managing wide-necked bifurcation aneurysms.^5^ This method involves deployment of two stents in a Y-configuration, which provides robust scaffolding that prevents coil prolapse into the parent vessels and ensures complete coverage of the aneurysm neck.^6,7^ Recent advancements include the pEGASUS stent system, a novel self-expanding open-cell stent designed specifically for neurovascular applications.^8^ The pEGASUS stent features an antithrombogenic hydrophilic polymer coating aimed at decreasing thromboembolic complications—a critical consideration in stent-assisted procedures. Initial studies have demonstrated promising results with the pEGASUS stent system, and indicated its potential efficacy and safety in the endovascular treatment of complex intracranial aneurysms.^8,9^

Despite these advancements, comprehensive data evaluating the long-term outcomes and procedural success rates of Y-SAC with pEGASUS stents remain limited. However, understanding these outcomes is essential for optimizing treatment strategies and improving patient prognoses. This retrospective observational study was aimed at assessing the effectiveness and safety of Y-SAC with pEGASUS stents in patients presenting with intracranial bifurcation aneurysms.

## Methods

### Study design and setting

This retrospective observational study was conducted at six neurovascular centers between July 13, 2021, and June 1, 2024. A retrospective review of electronic medical records identified patients treated with Y-stenting with pEGASUS stents. The study was approved by our institutional ethics committee (IRB: 24-180 RS). Because of the retrospective nature of the study, requirements for informed consent were waived. Patient confidentiality was ensured through anonymized data collection and storage.

### Participants

Patients were eligible for inclusion if they had ruptured or unruptured, wide-necked, saccular intracranial bifurcation aneurysms and underwent exclusively Y-SAC with two pEGASUS stents. Wide-necked aneurysms were defined as those with neck diameters ≥4 mm or dome-to-neck ratios <2.^10^ Patients were excluded if they had received prior aneurysm treatment, such as clipping.

### Procedures

All procedures were performed on patients under general anesthesia. Digital subtraction angiography and rotational angiography with 3D reconstructions were used to assess aneurysm morphology and parent artery anatomy. The Y-stenting technique involved the placement of two pEGASUS stents in a Y-configuration to provide neck coverage and prevent coil protrusion. The coiling system was used in all participants.

### Y-stent reconstruction

Endovascular treatment of wide-necked bifurcation aneurysms, especially those incorporating the origins of both daughter branches, remains challenging despite advances in balloon-assisted and single-stent techniques. This is particularly true for aneurysms at the middle cerebral artery (MCA) bifurcation, basilar apex, and carotid terminus.

At these bifurcations, a single stent may not provide sufficient neck coverage to protect both daughter vessels, and coil herniation into the unprotected branch remains a risk, even with balloon remodeling. The Y-stent technique offers a more robust reconstructive option by enabling coverage of the aneurysm neck while preserving flow in both daughter vessels.

**Figure 1.**
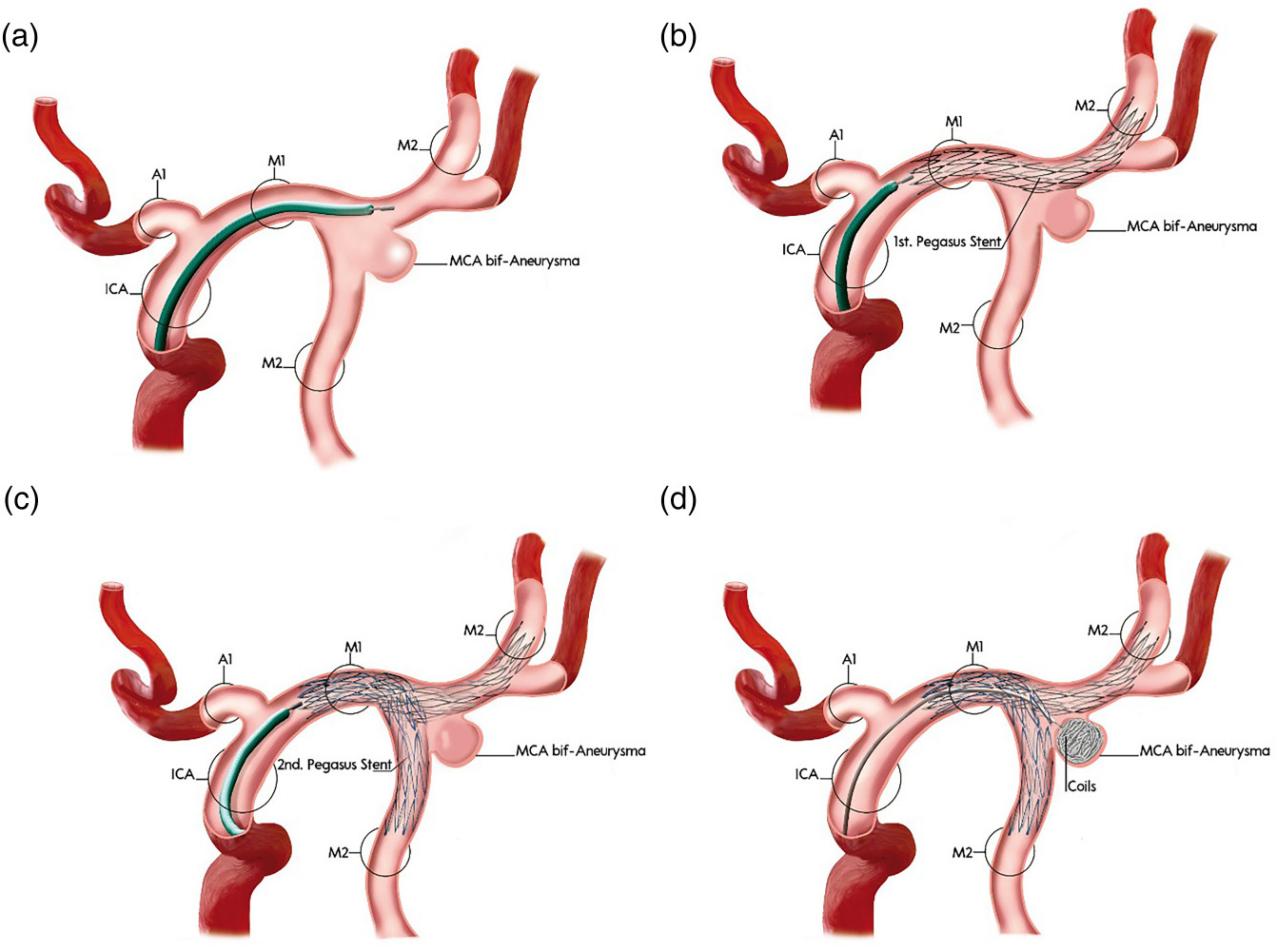
Stepwise schematic of Y-stent-assisted coil embolization of a wide-necked bifurcation aneurysm using the pEGASUS-HPC stent system.

**Figure 2.**
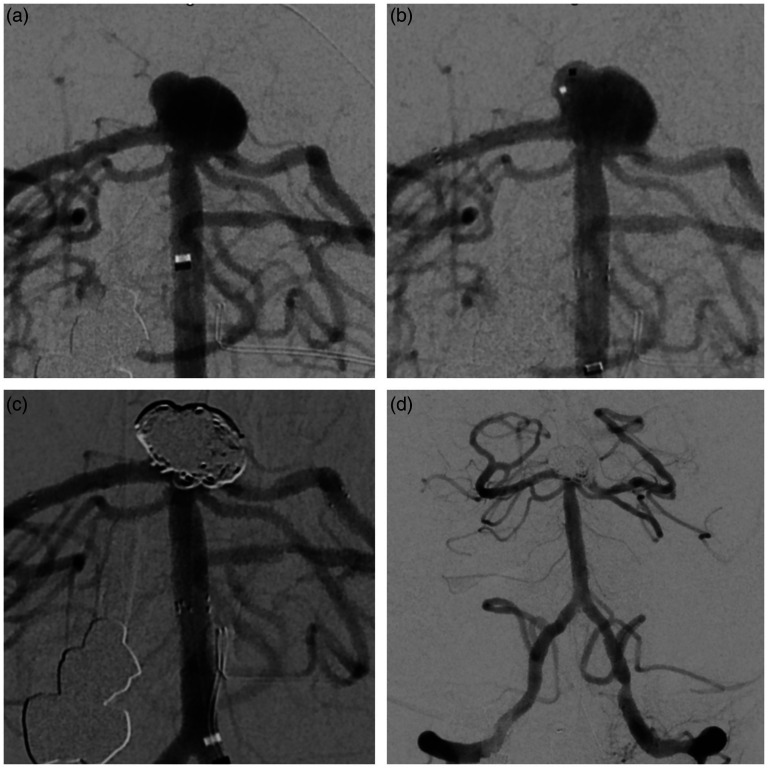
Endovascular treatment of a wide-necked basilar apex aneurysm using Y-stent-assisted coiling.

The following schematic (Figure 1) outlines the step-by-step application of the Y-stent technique using pEGASUS-hydrophilic polymer coating (HPC) stents for the treatment of a wide-necked MCA bifurcation aneurysm. In addition, a representative procedural case is presented (Figure 2), illustrating Y-stent-assisted coiling with pEGASUS stents, including pre- and post-interventional angiographic views. Supplementary Figures 1–6 provide additional procedural examples, highlighting anatomical variations and outcomes across different aneurysm locations and participating centers.

(a) A microcatheter is advanced through the ICA and M1 into the superior M2 branch. (b) The first pEGASUS stent is deployed from the superior M2 across the aneurysm neck into M1. (c) A second stent is placed through the struts of the first, from the inferior M2 into M1, completing the Y-configuration. (d) A microcatheter is advanced through the stent lumen into the aneurysm sac for coil embolization. ICA: internal carotid artery.

(a) Baseline DSA reveals a wide-necked saccular aneurysm at the basilar tip. (b) Two pEGASUS-HPC stents are deployed in a Y-configuration from the posterior cerebral arteries into the basilar artery. (c) Coil embolization is performed through the Y-stent construct, resulting in complete aneurysm packing. (d) Postprocedural DSA confirms preserved patency of both PCAs and complete aneurysm occlusion. DSA: digital subtraction angiography.

### Antiplatelet therapy protocols

Antiplatelet therapy in Y-SAC was individualized according to aneurysm status (ruptured vs. unruptured) and institutional protocols. The choice between prasugrel and ticagrelor was guided by physician preference and clinical context. Platelet function inhibition was confirmed in all cases through point-of-care testing, such as with VerifyNow assays, before stent deployment. For unruptured aneurysms, elective cases received dual antiplatelet therapy before the intervention to decrease thromboembolic risk: patients receiving a prasugrel-based regimen were given 100 mg aspirin and 10 mg prasugrel daily for at least 5–7 days pre-procedure, and this therapy continued for 3 months post-intervention; alternatively, a ticagrelor-based protocol comprised a 180 mg loading dose on the day of the procedure, followed by 100 mg aspirin once daily and 90 mg ticagrelor twice daily for 3 months. Platelet inhibition testing was optionally performed with both regimens. In ruptured aneurysms, a relatively conservative strategy was used to balance hemorrhagic risk with thromboembolic protection: patients received a 60-mg loading dose of prasugrel followed by 10 mg daily, and repeat loading was considered if platelet inhibition was suboptimal.

### Data collection

Data collected retrospectively from medical records included demographics, aneurysm characteristics, procedure-related details, and imaging follow-up at 6 and 12 months. The data were anonymized and stored in an Excel database, whereas imaging assessments were conducted with the routine picture archiving and communication system (PACS) system.

Aneurysm occlusion was evaluated with the modified Raymond–Roy classification (MRRC), in which class I indicated complete occlusion with no filling of the aneurysm, class II indicated a neck remnant with more than 90% occlusion, and class III indicated incomplete occlusion with filling of the aneurysm lumen. Follow-up imaging with digital subtraction angiography, MRI, or CT was performed at 6 and 12 months.

### Statistical analysis

Baseline characteristics, procedural success, and clinical outcomes were summarized with descriptive statistics. Continuous variables are reported as means with standard deviations or medians with interquartile ranges, depending on normality. Categorical variables are presented as frequencies and percentages. Statistical analyses were conducted in Jamovi Software, with a significance level set at *P* < .05.

## Results

### Demographic and clinical characteristics

The study included 40 patients with intracranial aneurysms, with a mean age of 61.6 ± 9.4 years. The cohort comprised 24 women (60.0%) and 16 men (40.0%). Aneurysms were most frequently located in the anterior communicating artery (32.5%), followed by the basilar artery (27.5%), middle cerebral artery (22.5%), internal carotid artery (ICA) (10.0%), and posterior communicating artery (7.5%). Anterior circulation aneurysms were more prevalent (67.5%) than posterior circulation aneurysms (32.5%). Whereas 22.5% of aneurysms were on the left side, 17.5% were on the right side; moreover, 60.0% were located in the basilar artery and anterior communicating artery. All aneurysms were saccular in nature, and no prior treatments were recorded. Ruptured aneurysms were identified in 17.5% of cases, whereas 82.5% remained unruptured. The mean aneurysm neck width was 4.6 ± 1.7 mm, the mean sack width was 5.7 ± 2.8 mm, and the mean sack depth was 5.1 ± 3.2 mm. The dome-to-neck ratio averaged 1.4 ± 0.6 (Table 1).

**Table 1.** Demographic, clinical, and radiological characteristics of patients with intracranial aneurysms.

Parameters		*N*(%)/mean ± SD
Age		61.6 ± 9.4
Gender	Female	24 (60.0%)
Aneurysm location	PCOM	3 (7.5%)
	AcomA	13 (32.5%)
	BA	11 (27.5%)
	ACI	4 (10.0%)
	MCA	9 (22.5%)
Anterior vs. posterior	Anterior	27 (67.5%)
Aneurysm side	Left	9 (22.5%)
	Right	7 (17.5%)
	BA and AcomA	24 (60.0%)
Aneurysm type	Saccular	40 (100.0%)
Previous treatment	No	40 (100.0%)
Ruptured aneurysm	Yes	7 (17.5%)
Aneurysm neck width (mm)		4.6 ± 1.7
Aneurysm sack width (mm)		5.7 ± 2.8
Aneurysm sack depth (mm)		5.1 ± 3.2
Dome to neck ratio		1.4 ± 0.6

### Procedural characteristics

The procedural and device characteristics of patients undergoing stent-assisted aneurysm treatment are detailed in Table 2. The mean diameter of the first pEGASUS device was 3.8 ± 0.5 mm, and most cases received a 3.5 mm (67.5%) or 4.5 mm (32.5%) stent. The mean length of the first pEGASUS device was 23.3 ± 3.7 mm, and 50.0% of cases received a 20 mm stent, 35.0% received a 25 mm stent, and 15.0% received a 30 mm stent. Similarly, for the second pEGASUS device, the mean diameter was 3.8 ± 0.5 mm, and 67.5% of patients received a 3.5 mm stent, whereas 32.5% received a 4.5 mm stent. The mean length of the second device was 23.5 ± 3.4 mm, and most patients received 25 mm (60.0%) or 20 mm (27.5%) stents, whereas fewer received 30 mm (7.5%) or 15 mm (5.0%) stents. The transfemoral approach was predominantly used (95.0%), and a triaxial system was used in 92.5% of cases.

**Table 2.** Procedural and device characteristics in patients undergoing stent-assisted aneurysm treatment.

Parameters		Mean ± SD/*N*(%)
1st pEGASUS device diameter (mm)	Mean ± SD	3.8 ± 0.5
	3.5 mm	27 (67.5%)
	4.5 mm	13 (32.5%)
1st pEGASUS device length (mm)	Mean ± SD	23.3 ± 3.7
	20 mm	20 (50.0%)
	25 mm	14 (35.0%)
	30 mm	6 (15.0%)
2nd pEGASUS device diameter (mm)	Mean ± SD	3.8 ± 0.5
	3.5 mm	27 (67.5%)
	4.5 mm	13 (32.5%)
2nd pEGASUS device length (mm)	Mean ± SD	23.5 ± 3.4
	15.0 mm	2 (5.0%)
	20.0 mm	11 (27.5%)
	25.0 mm	24 (60.0%)
	30.0 mm	3 (7.5%)
Stenting approach	Transfemoral	38 (95.0%)
Used system	Triaxial	37 (92.5%)
Proximal artery segment of the 1st and 2nd pEGASUS	ICA	7 (17.5%)
	V4	1 (2.5%)
	A1	13 (32.5%)
	BA	10 (25.0%)
	M1	9 (22.5%)
Proximal artery measurements of the 1st and 2nd pEGASUS		2.9 ± 0.6
Daughter artery segment of the 1st device	A1	1 (2.5%)
	A2	14 (35.0%)
	BA	1 (2.5%)
	M1	5 (12.5%)
	M2	9 (22.5%)
	PCA	7 (17.5%)
	P1	3 (7.5%)
Distal artery measurements of the 1st pEGASUS		2.0 ± 0.5
Daughter artery segment of the 2nd pEGASUS	A1	3 (7.5%)
	A2	12 (30.0%)
	BA	1 (2.5%)
	M1	2 (5.0%)
	M2	9 (22.5%)
	PCA	7 (17.5%)
	P1	3 (7.5%)
	Pcom	3 (7.5%)
Daughter artery measurements of the 2nd pEGASUS		1.8 ± 0.4
Days of hospitalization mean ± SD/median (IQR)		4.2 ± 5.2/3 (2–3)

ICA: internal carotid artery.

Regarding arterial placement, the most common proximal artery segments for the first and second pEGASUS devices were the A1 segment (32.5%), basilar artery (25.0%), M1 segment (22.5%), internal carotid artery (17.5%), and V4 (2.5%). The mean proximal artery measurement was 2.9 ± 0.6 mm. For the first pEGASUS device, the most common daughter artery segment was A2 (35.0%) and was followed by M2 (22.5%), the posterior cerebral artery (17.5%), M1 (12.5%), P1 (7.5%), A1 (2.5%), and the basilar artery (2.5%); the mean distal artery measurement was 2.0 ± 0.5 mm. Similarly, for the second pEGASUS device, A2 remained the most frequently involved daughter artery segment (30.0%) and was followed by M2 (22.5%), the posterior cerebral artery (17.5%), A1 (7.5%), P1 (7.5%), Pcom (7.5%), M1 (5.0%), and the basilar artery (2.5%); the mean daughter artery measurement was 1.8 ± 0.4 mm. The median hospital stay was 3 days (interquartile range: 2–3 days), and the mean duration was 4.2 ± 5.2 days.

### Procedural outcomes

**Figure 3.**
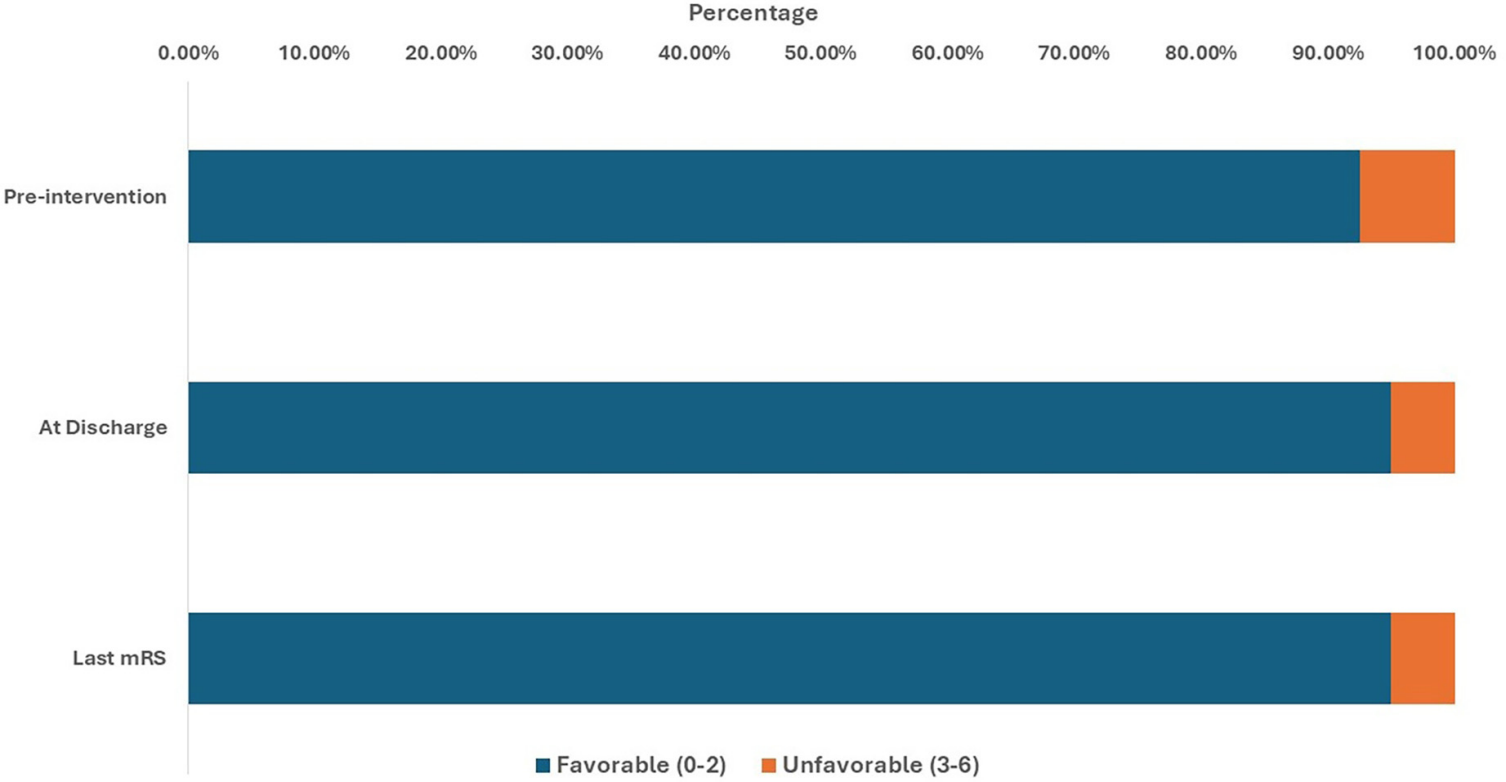
MRS of the included participants pre-intervention, at discharge, and at the endpoint.

Before the intervention, 92.5% of patients had a favorable functional status, whereas 7.5% had unfavorable status. At discharge, the proportion of favorable outcomes slightly increased, to 95.0%, and unfavorable outcomes correspondingly decreased to 5.0% (Figure 3). Among patients with ruptured aneurysms, the percentage of unfavorable outcomes decreased from 42.9% before the intervention to 28.6% at discharge and at the last follow-up. Retreatment was required in only one patient (2.5%).

Immediately post-intervention, all patients (100%) had MRRC I occlusion. At the 3–6 month follow-up, two patients (5%) were recorded as deceased. All patients who were investigated at follow-up visits (*n* = 38, 95%) maintained MRRC I occlusion. By the 6–12 month follow-up, MRRC I occlusion was observed in 92.5% of patients, whereas 2.5% had MRRC II. At the last follow-up, 95% of patients had MRRC I occlusion.

## Discussion

The management of intracranial aneurysms, particularly those with wide necks or complex morphologies, has historically presented major challenges in neurointervention. Conventional endovascular coiling, although minimally invasive, often faces limitations such as coil instability and elevated risk of aneurysm recurrence, particularly in wide-necked aneurysms.^11^ Stent-assisted coiling techniques developed to address these challenges provide structural support to prevent coil prolapse and promote durable aneurysm occlusion.^12^ The Y-SAC's robust scaffolding ensures complete coverage of the aneurysm neck and prevents coil migration into parent vessels.^13^ However, the use of intracranial stents necessitates antiplatelet therapy to mitigate thromboembolic risks and therefore can complicate treatment, particularly in cases of ruptured aneurysms.^14^ The pEGASUS stent system introduces an innovative solution to these challenges. Designed for optimal adaptation to various vessel anatomies, the pEGASUS stent is available with an antithrombogenic HPC.^8,15^ This HPC surface modification significantly decreases thrombogenic complications, thereby potentially minimizing the need for prolonged dual antiplatelet therapy and enhancing patient safety.^16^ By combining the structural advantages of Y-SAC with the thromboresistant properties of the HPC-coated pEGASUS stent, this approach is aimed at improving the safety and efficacy of endovascular treatment for complex intracranial aneurysms.

Compared with other widely used stent systems, such as Neuroform and low-profile visualized intraluminal support (LVIS), the pEGASUS HPC stent offers several potential advantages in Y-SAC.^17,18^ pEGASUS features an open-cell architecture that enhances conformability and facilitates access to side branches during Y-configuration deployment. Compared with LVIS, a braided stent used with Y-stenting requiring microcatheter jailing,^19^ the laser-cut pEGASUS does not require jailing with lower thrombogenicity due to antithrombogenic hydrophilic polymer coating. Unlike with Neuroform without coating,^18^ HPC coating of pEGASUS might decrease the need for prolonged dual antiplatelet therapy and has been associated with diminished thrombembolic events in some studies.^16^ Although direct head-to-head comparisons are lacking, the low rate of periprocedural thromboembolic events observed with pEGASUS supports the potential clinical benefits of this stent in Y-SAC configurations. Future prospective studies should investigate these comparative aspects in controlled settings.

Y-SAC has been extensively studied for the treatment of wide-necked bifurcation intracranial aneurysms and has yielded favorable outcomes across various investigations. In a study by Lee et al., in 20 patients who underwent Y-SAC, a 95% complete occlusion rate was achieved immediately post-procedure, and a 5% recurrence rate was observed during follow-up.^20^ Similarly, Aydin et al. have reported a 97.4% complete occlusion rate immediately post-procedure and a 2.6% recurrence rate at long-term follow-up, among 38 patients treated with T-stent-assisted coiling, a variant of Y-SAC.^21^ Melber et al. have evaluated the use of low-profile Acandis Acclino stents in 40 patients and reported a 92.5% complete occlusion rate immediately post-procedure and a 7.5% recurrence rate at long-term follow-up.^22^ A systematic review and meta-analysis by Gunkan et al., encompassing 15 studies in a total of 438 patients, has reported a 93.2% complete occlusion rate immediately post-procedure and a 6.8% recurrence rate during follow-up.^23^

In this retrospective observational study, we evaluated the effectiveness and safety of Y-SAC with pEGASUS stents in treating intracranial bifurcation aneurysms. Our findings demonstrated high rates of aneurysm occlusion, low complication rates, and favorable clinical outcomes either similar to or surpassing results from recent studies using similar techniques.​

Our study achieved immediate complete occlusion (MRRC class I) in 100% of cases post-intervention. At the 6–12 month follow-up, 92.5% of patients maintained MRRC class I occlusion and 2.5% exhibited class II occlusion. These results are consistent with those in a study by Pielenz et al., in which immediate complete occlusion occurred in 83.3% of patients treated with the pEGASUS stent system, and 90.9% maintained complete occlusion at a mean follow-up of 7.4 months.^8^ Similarly, a study using the LEO Baby stent has reported immediate occlusion rates of 95.5% and maintenance of complete occlusion at 6 months in 94.6% of patients.^24^ These outcomes are consistent with those from the aforementioned studies, thereby underscoring the effectiveness of Y-SAC across various stent systems.

In our cohort, 95% of patients had favorable functional outcomes at discharge, and no procedure-related mortality was observed. The overall complication rate was 4.8%, and one patient (2.5%) required retreatment. These findings are comparable to those of Rodriguez Caamaño et al., who have reported no treatment-related mortality and a 2.5% rate of major ipsilateral strokes with the Y-SAC technique with double Neuroform stents.^25^ Similarly, Piotin et al. have observed permanent neurological procedure-related complications in 7.4% of procedures with stents, compared with 3.8% without stents, thus highlighting the safety profile of stent-assisted coiling techniques.^26^

The pEGASUS stent system's antithrombogenic HPC is designed to decrease thromboembolic complications, a critical consideration in stent-assisted procedures.^15^ In our study, we observed no periprocedural thromboembolic events, in agreement with the findings of Boxberg et al., who have reported no periprocedural complications in patients treated electively with the pEGASUS stent system.^9^ In contrast, studies using other stent systems, such as the LEO Baby, have reported overall complication rates of 4.8%, including one patient death during post-procedural follow-up.^24^ These comparisons suggest that the pEGASUS stent system might offer a favorable safety profile in Y-SAC procedures.​

### Clinical implications and future directions

The integration of Y-SAC with pEGASUS stents has substantial clinical implications for the treatment of complex intracranial aneurysms. Our study demonstrated high rates of durable aneurysm occlusion and favorable clinical outcomes, suggesting the safety and effectiveness of this approach. The antithrombogenic HPC of the pEGASUS stent might avoid thromboembolic complications and potentially minimize the need for prolonged dual antiplatelet therapy. ​Future research should focus on long-term outcomes and direct comparisons with other stent systems to validate these findings and establish optimal treatment protocols for patients with wide-necked bifurcation aneurysms.

### Limitations

The retrospective nature of our study and the relatively small sample size limit the generalizability of our findings. Because of the limited sample size, we were unable to perform subgroup or sensitivity analyses to assess potential differences in outcomes by antiplatelet therapy type. Additionally, the follow-up period was limited to 6–12 months, and longer-term outcomes remain to be evaluated. Future prospective studies with larger cohorts and extended follow-up periods are warranted to confirm the durability and safety of Y-SAC with pEGASUS stents.​

## Conclusion

The findings of this study support the efficacy and safety of Y-SAC with pEGASUS stents for the treatment of wide-necked intracranial bifurcation aneurysms. Our results demonstrated high rates of immediate and sustained aneurysm occlusion, with favorable functional outcomes and minimal complications. The integration of pEGASUS stents, featuring an antithrombogenic HPC, might contribute to decreasing thromboembolic risk and enhancing long-term safety. Future investigations should also explore the roles of alternative stent designs and adjunctive therapies in optimizing outcomes for patients with complex intracranial aneurysms.

## Supplemental Material

sj-docx-1-ine-10.1177_15910199251360143 - Supplemental material for Y-stent-assisted coiling with pEGASUS stents for intracranial bifurcation aneurysms: A multi-center retrospective study Supplemental material, sj-docx-1-ine-10.1177_15910199251360143 for Y-stent-assisted coiling with pEGASUS stents for intracranial bifurcation aneurysms: A multi-center retrospective study by Abdallah Aburub, Ali Khanafer, Zakarya Ali, Mohammad Almohammad, Oussama Dob, Mete Dadak, Lars Timmermann, Ole Simon, Anja Gerstner, Mariana Gurschi, Yashar Aghazadeh, Christopher Nimsky, Benjamin Saß, Hans Henkes, André Kemmling and Stephan Felber in Interventional Neuroradiology
