# Supplementary material for: Y-stent-assisted coiling with pEGASUS stents for intracranial bifurcation aneurysms: A multi-center retrospective study
Source: Interv Neuroradiol. 2025 Aug 1:15910199251360143. Online ahead of print. doi: 10.1177/15910199251360143 (PMC12316673; doi:10.1177/15910199251360143)
Supplement: sj-docx-1-ine-10.1177_15910199251360143 - Supplemental material for Y-stent-assisted coiling with pEGASUS stents for intracranial bifurcation aneurysms: A multi-center retrospective study [file sj-docx-1-ine-10.1177_15910199251360143.docx]

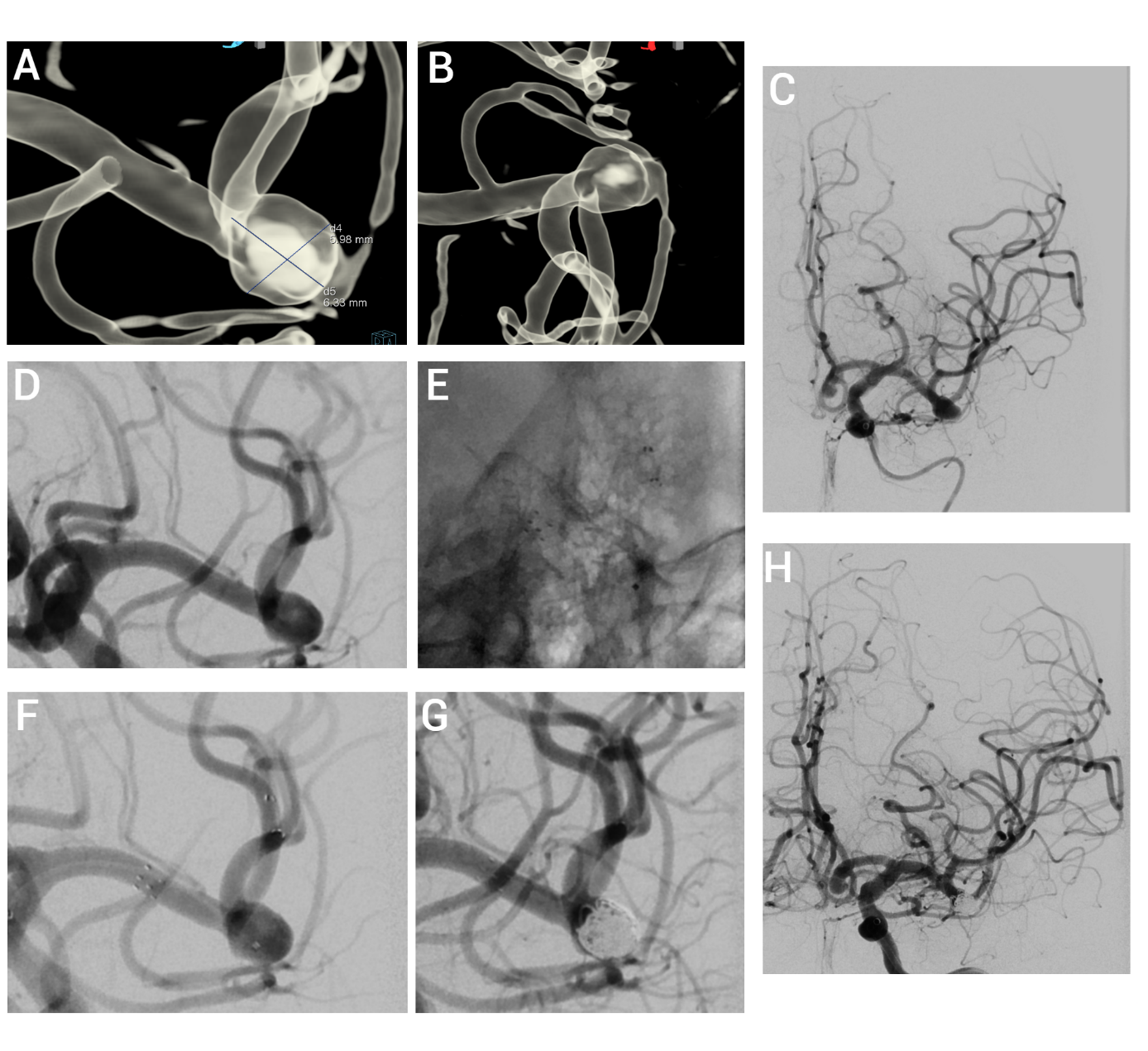
**Supplementary Figure 1:** Endovascular treatment of a wide-necked middle cerebral artery (MCA) bifurcation aneurysm using double stent-assisted coiling.

(A, B) 3D rotational angiography demonstrates a wide-necked saccular aneurysm at the MCA bifurcation. (C) Baseline digital subtraction angiography (DSA) in overview projection confirms aneurysm morphology and vascular anatomy. (D) Working projection with magnification is selected for optimal visualization of aneurysm neck and daughter vessels. (E) Native fluoroscopy after deployment of two stents in crossing Y-configuration. (F) DSA confirms correct positioning of both stents and reconstruction of the bifurcation with preserved flow. (G) Final angiographic result after coil embolization shows complete aneurysm occlusion. (H) Postprocedural overview DSA confirms stable occlusion and preserved perfusion of both MCA branches.


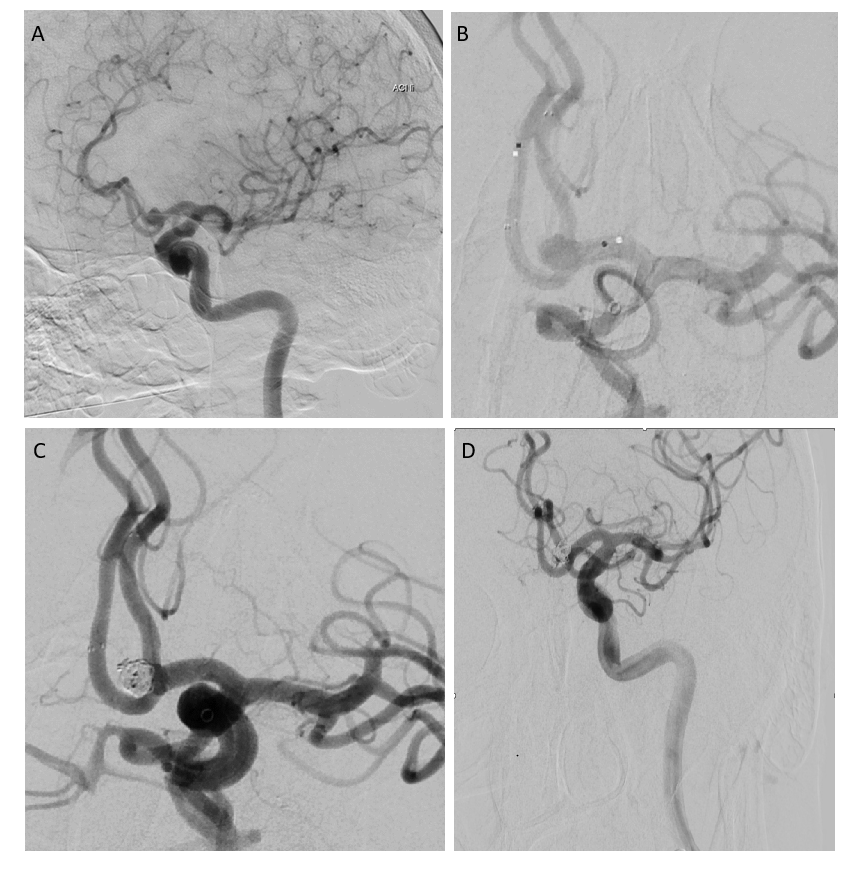
**Supplementary Figure 2:** Endovascular treatment of a wide-necked right MCA bifurcation aneurysm using Y-stent-assisted coiling.
(A) Baseline DSA reveals a wide-necked saccular aneurysm at the right MCA bifurcation. (B) Two pEGASUS-HPC stents are deployed in a Y-configuration from the superior and inferior M2 branches into the M1 segment. (C) Coil embolization is performed through the Y-stent construct, resulting in complete aneurysm packing. (D) Postprocedural DSA confirms preserved patency of both M2 branches and complete aneurysm occlusion.

**
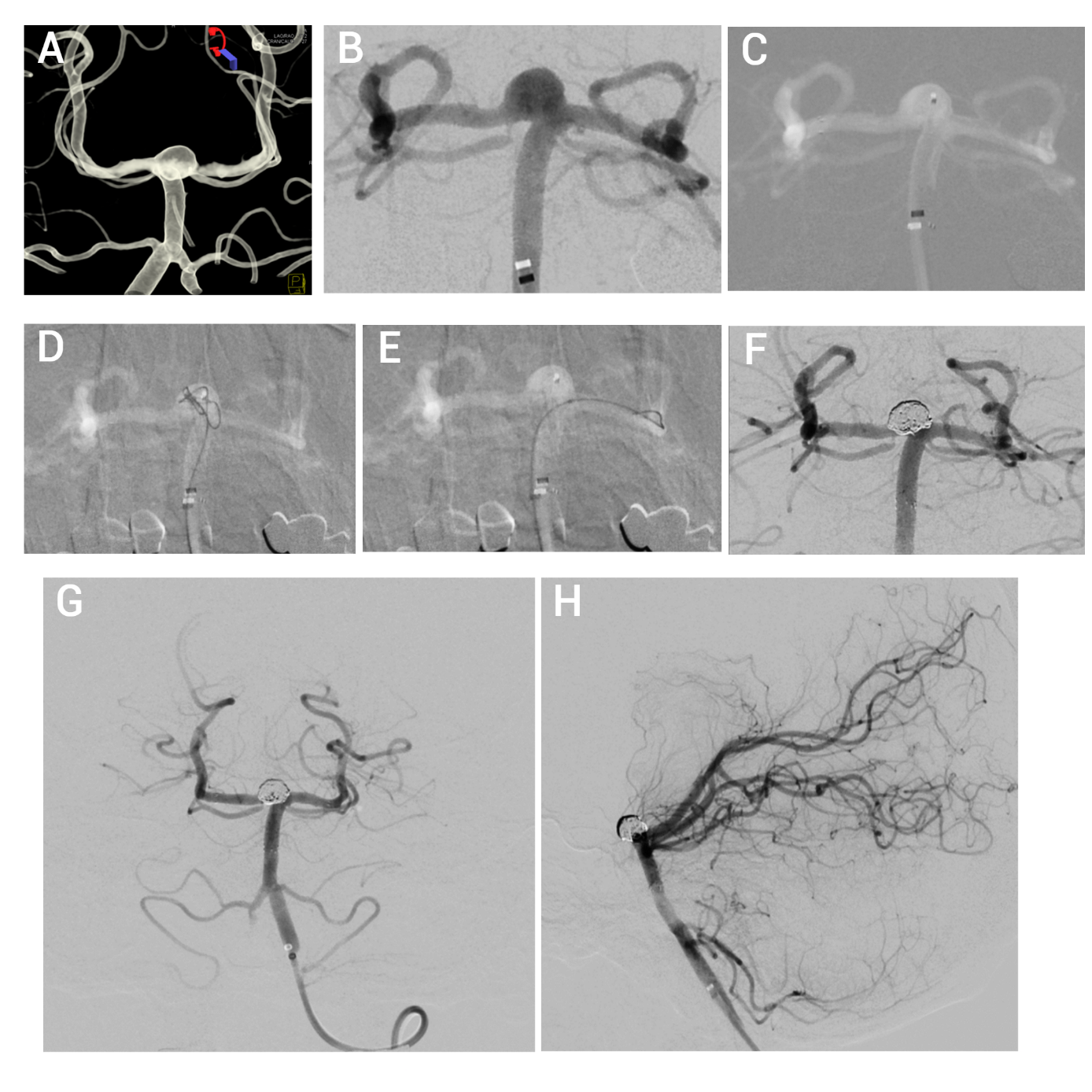
Supplementary Figure 3:** Endovascular treatment of a wide-necked basilar apex aneurysm using Y-stent-assisted coiling.

(A, B) Baseline 3D rotational angiography and DSA demonstrate a wide-necked saccular aneurysm at the basilar tip. (C) After deployment of the first pEGASUS stent from the basilar artery into the right posterior cerebral artery (PCA). (D) Initial coiling attempt reveals coil herniation into the left PCA. (E) A second pEGASUS stent is deployed from the basilar artery into the left PCA through the struts of the first, completing the Y-configuration. (F) Final coil embolization is performed within the Y-stent construct. (G, H) Postprocedural DSA confirms complete aneurysm occlusion with preserved patency of both PCAs.

**
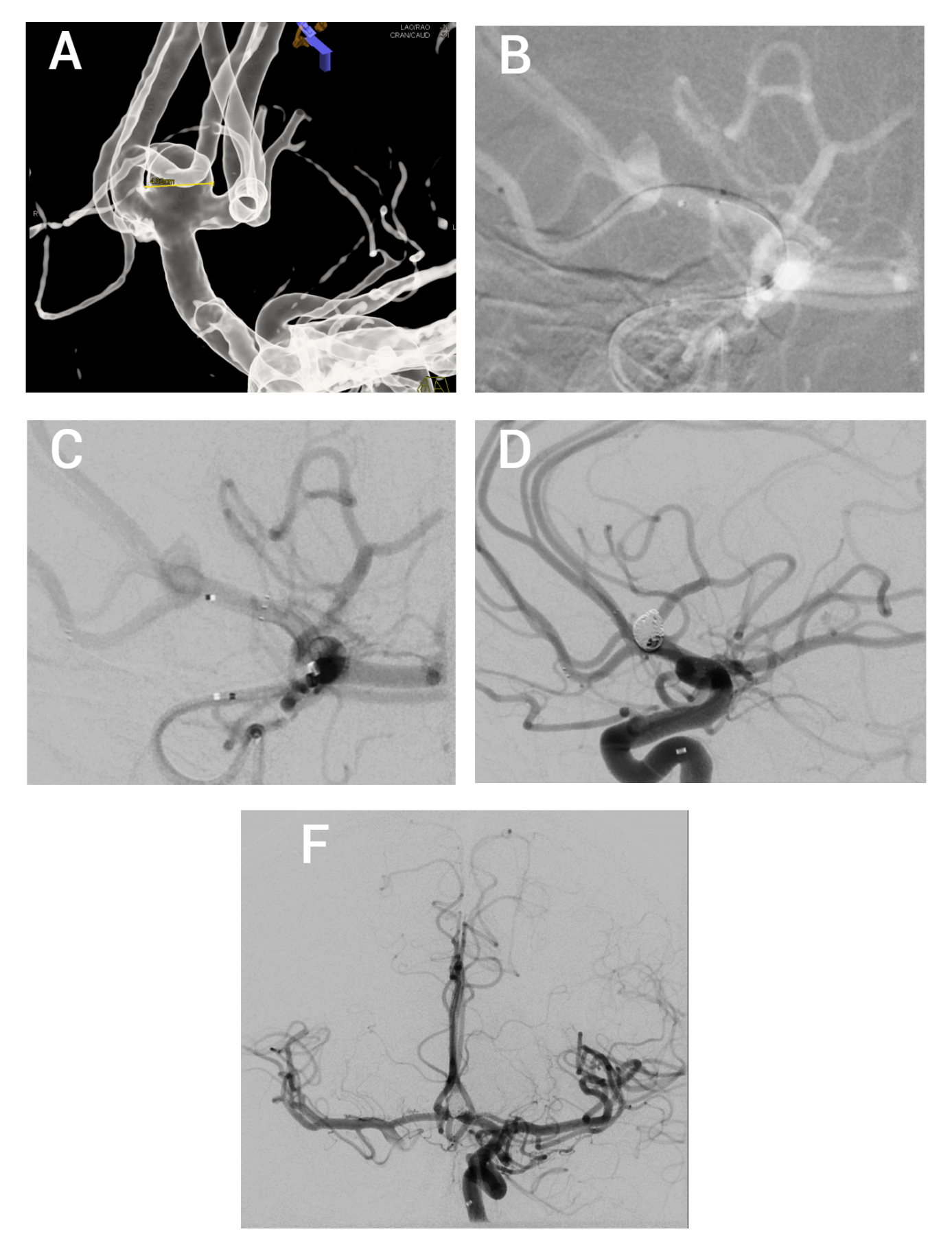
Supplementary Figure 4:** Endovascular treatment of a wide-necked anterior communicating artery (Acom) aneurysm using Y-stent-assisted coiling with pEGASUS-HPC stents.

(A) 3D rotational angiography shows a wide-necked Acom aneurysm involving both A2 segments. (B) Microcatheter positioning into the left A2 segment through the right A1 for initial stent deployment. (C) First pEGASUS-HPC stent deployed from the left A2 to the right A1 segment. (D) Second stent deployed from the right A2 into the right A1 through the struts of the first, completing the Y-configuration, followed by coil embolization. (F) Postprocedural DSA confirms complete aneurysm occlusion and preserved flow in both A2 segments.

**
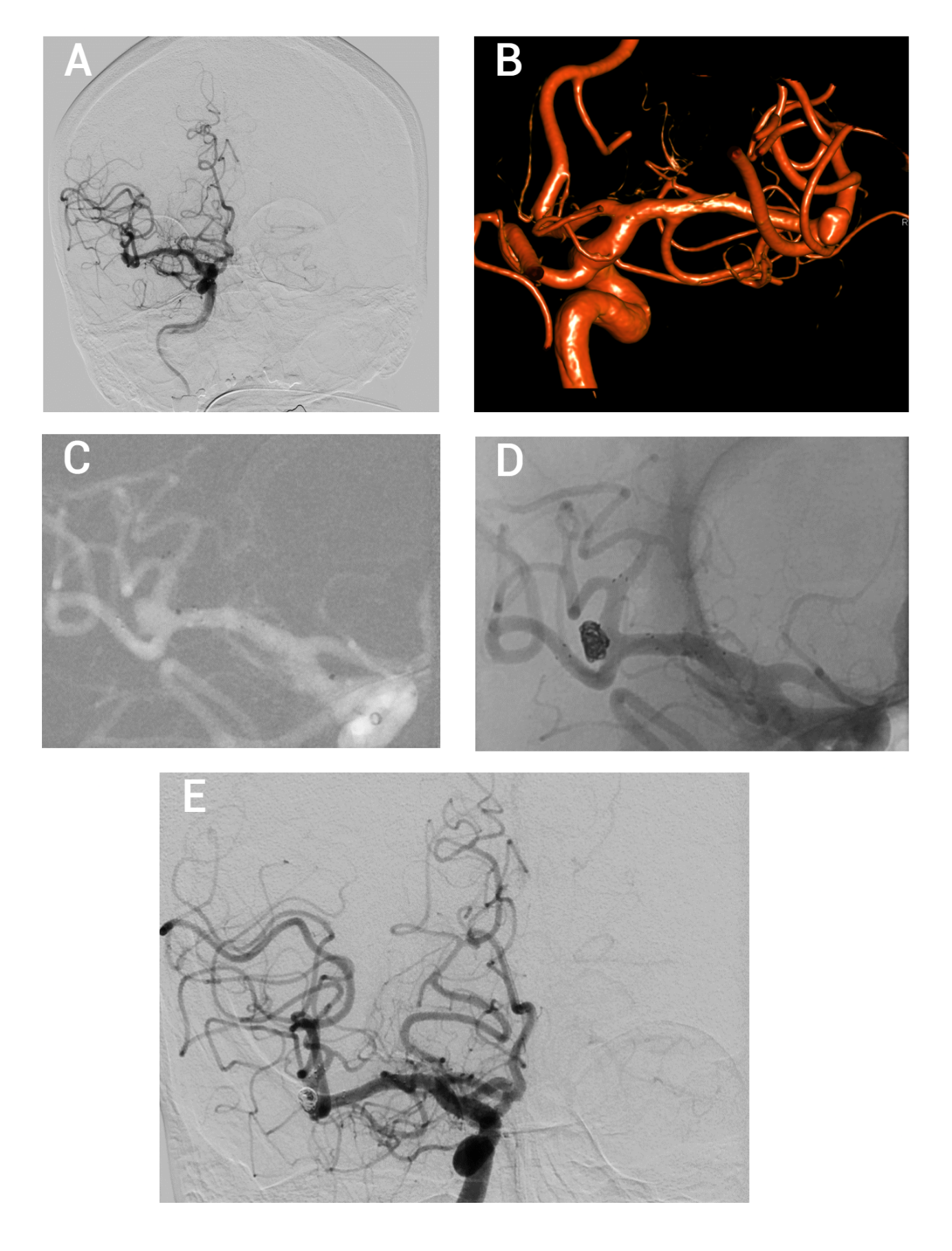
Supplementary Figure 5:** Endovascular treatment of a wide-necked right MCA bifurcation aneurysm using Y-stent-assisted coiling with pEGASUS-HPC stents.

(A) Baseline DSA reveals a wide-necked saccular aneurysm at the right MCA bifurcation. (B) 3D rotational angiography confirms complex bifurcation anatomy involving both M2 branches. (C) Two pEGASUS-HPC stents are implanted in Y-configuration from the superior and inferior M2 branches into the M1 segment.. (D) Coil embolization is performed through the Y-stent construct. (E) Final DSA demonstrates complete aneurysm occlusion with preserved patency of both M2 branches.


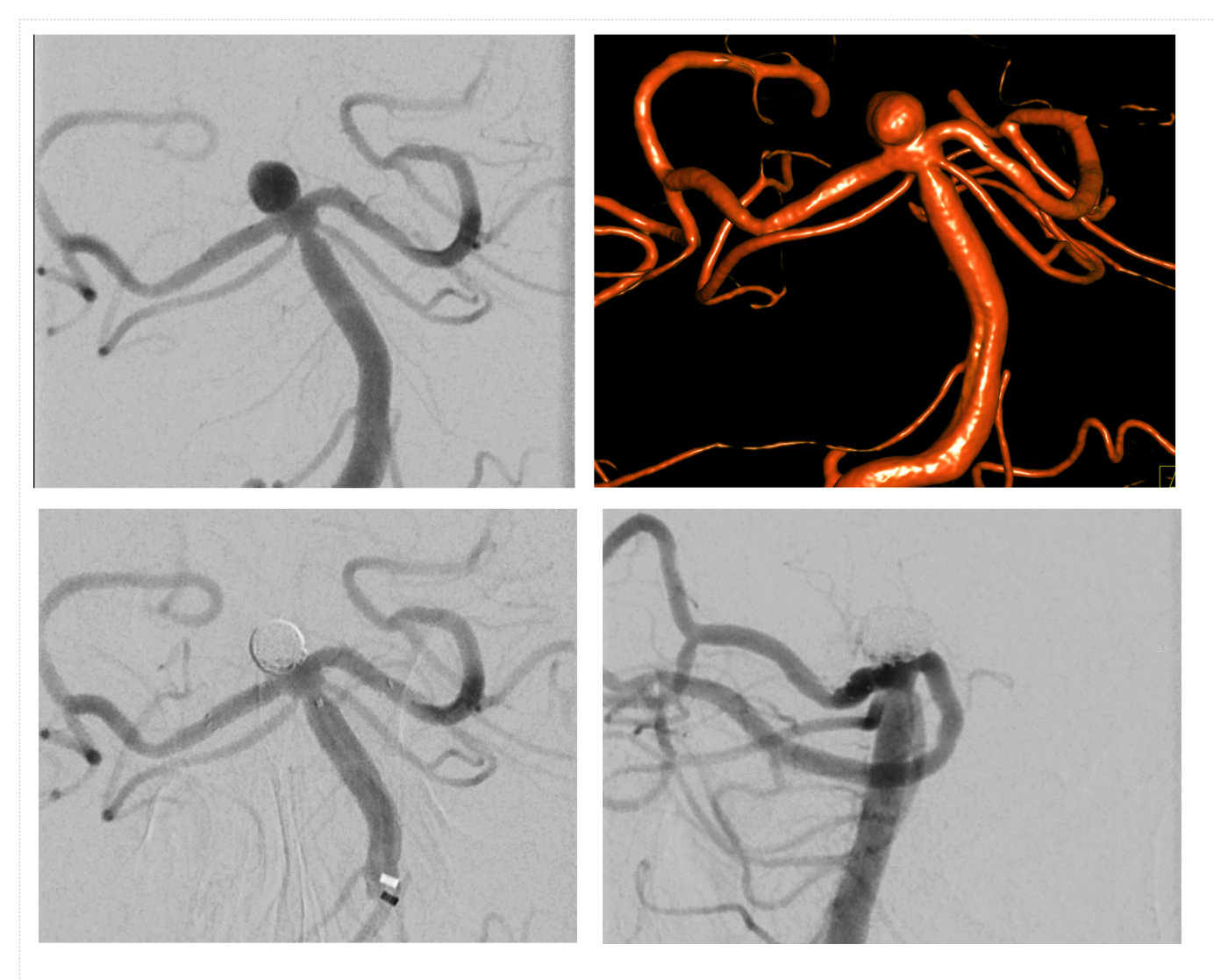
**Supplementary Figure 6:** Endovascular treatment of a wide-necked basilar tip aneurysm using stent-assisted coiling.
(A) Baseline digital subtraction angiography (DSA) shows a wide-necked saccular aneurysm at the basilar tip involving the origins of both posterior cerebral arteries (PCAs). (B) 3D rotational angiography confirms aneurysm morphology and vessel anatomy. (C, D) Postinterventional DSA demonstrates dense coil packing within the aneurysm sac and confirms complete aneurysm occlusion with preserved patency of both PCAs.
